# Supplementary material for: Insight into the protein solubility driving forces with neural attention
Source: PLoS Comput Biol. 2020 Apr 30;16(4):e1007722. doi: 10.1371/journal.pcbi.1007722 (PMC7217484; doi:10.1371/journal.pcbi.1007722)
Supplement: S1 Text — PDF file containing additional analyses. (PDF) [file pcbi.1007722.s001.pdf]

Supplementary Material

S1 Correlation between the attention and biophysical values

Table A1: Table showing the Pearson's correlation between the attention, prediction and solubility profiles (attention × prediction) with various biophysical values and prediction values with biophisical propensity scales

Value	Hydro	Polairty	Charge	Volume	Alpha	Beta
Attention	-0.041	0.001	0.047	0.001	0.012	0.001
Prediction	0.067	0.113	0.019	-0.246	0.169	0.064
Att × Pred	0.011	0.009	-0.023	-0.033	0.01	0.015

S2 Mutations with highest ΔS on O26734

('V:0:M', -0.3779628276824951) ('V:0:F', -0.3599933087825775) ('D:11:T', -0.33895576000213623) ('V:0:W', -0.331144243478775) ('V:0:N', -0.3297457993030548) ('V:0:R', -0.3012681007385254) ('E:18:T', -0.2916121184825897) ('V:0:L', -0.29109594225883484) ('V:0:I', -0.2907434105873108) ('V:0:Y', -0.28444716334342957) ('V:0:K', -0.27677634358406067) ('E:8:M', -0.2691635489463806) ('V:0:T', -0.26681238412857056) ('V:0:G', 0.24836838245391846) ('I:1:F', -0.24678555130958557) ('V:0:Q', -0.24508073925971985) ('E:18:Q', -0.24361136555671692) ('E:8:N', -0.24200883507728577) ('M:3:Y', -0.23842570185661316) ('D:4:A', -0.2349146008491516) ('E:8:L', -0.2348630130290985) ('D:4:H', -0.2313416600227356) ('V:0:P', -0.22996610403060913) ('I:1:W', -0.2295425534248352) ('P:21:M', -0.22810333967208862) ('E:8:I', -0.21945106983184814) ('M:3:A', -0.21550941467285156) ('V:9:T', -0.21287566423416138) ('D:11:Q', -0.21283787488937378) ('D:4:M', -0.20988613367080688) ('E:18:I', -0.20656836032867432) ('M:3:W', -0.201288640499115) ('E:8:D', -0.20068126916885376) ('V:0:E', -0.1991795301437378) ('V:0:D', 0.19838804006576538) ('E:8:T', -0.19740581512451172) ('D:4:F', -0.19348138570785522) ('T:2:I', -0.18687599897384644) ('V:19:Q', -0.18604910373687744) ('V:0:H', -0.18581873178482056) ('E:8:Q', -0.18208098411560059) ('I:17:Q', -0.17605316638946533) ('M:3:H', -0.175997793674469) ('T:2:W', -0.17561686038970947) ('V:0:C', -0.1747894287109375) ('C:5:H', -0.17460280656814575) ('E:8:R', -0.1740822196006775) ('M:3:G', -0.1711832880973816) ('I:1:G', -0.16442817449569702) ('T:2:A', -0.16376203298568726)
