# Supplementary material for: Insight into the protein solubility driving forces with neural attention
Source: PLoS Comput Biol. 2020 Apr 30;16(4):e1007722. doi: 10.1371/journal.pcbi.1007722 (PMC7217484; doi:10.1371/journal.pcbi.1007722)
Supplement: Attachment — Submitted filename: answersReviewers.pdf [file pcbi.1007722.s011.pdf]

Reviewer #1:

This is an interesting manuscript in the burgeoning field of protein solubility prediction. It is based on machine learning models, and produces results in line with other available methods. The authors describe their use of ML methods as novel in the field and this may be the case, I am afraid that I am not expert in ML methods. What I regard as novel are a couple of observations made in the manuscript, and that is where I would like to see more work carried out to clarify the results.

First, a statement is made to the effect that the predictions / model are not based on the commonly used biophysical properties (of amino acids). But, I do not see how this can be asserted if amino acids themselves are central to the prediction scheme - as I see it, sequence is the input to the training algorithm.

By stating that we do not use the “biophysical propensity scales” we mean that we do not use them ***explicitly***, as input to our model. This means that we do not use the “classic” physicochemical scales (e.g. hydropathy (Kyte and Doolittle 1982), polarity (Stryer, 1995), side-chain volume (Zamyatin, 1972), etc.) that associate each aminoacid with a particular numeric value to compute the input features for our method.

These scales are readily available in databases such as Aaindex (https://www.genome.jp/aaindex/) and are often used as input features in ML methods, since these algorithms need as input a numeric representation of the residues in the protein sequence, and the propensity scales provide a relatively easy way to compute these features. Notwithstanding their *apparent* straightforwardness, there is more to it when they are used as input features in ML methods. Recently, we published a study showing that the role of those scales as input features in ML methods has quite unexpected limitations (https://www.nature.com/articles/s41598-019-53324-w), showing that embeddings are a more straightforward (and agnostic) way to actually obtain the expected results. For this reason, our policy is to avoid the *explicit* using these scales to build feature vectors, and in the Neural Network (NN) context we prefer to use embeddings.

Embeddings, such as the ones used in SKADE, are just additional trainable weights that allow the model itself to learn the “optimal” numeric representation for the 20 aminoacids during training. This allows the model to learn the numeric description for each aminoacid type that is optimal for the task at hand (the protein solubility prediction in our case), instead of being forced to use the fixed propensity scales chosen as features by the model creator.

By stating that we do not use biophysical propensity scales we do not mean that biophysical properties of aminoacids are not relevant for protein solubility. Our statement refers purely to the ML method input details.

Second, and of the most interest to me, is the observation that N-t and C-t seem to possess (Fig. 2) more or less all the predictive power. I would not be surprised to find a tendency towards greater predictive power, but a flat line (Fig. 2) over the central 70% of the sequence I find amazing. Is it possible that the method is learning sequences (i.e. homology) and that this is best expressed at NT and CT when sequences of variable lengths are mapped to percentiles?

My own speculation is probably wrong, but I feel that the authors need more work here. e.g. What happens if the standard bioinformatics methods for reducing to non-redundant training set is used?

We also were surprised by this finding, and for this reason we ran additional analyses. Now we extended the comparison between DeepSolS1 and SKADE for what concerns the preferential use of the N- and C- termini with respect to the central region of the sequence. We now show that also DeepSolS1, which has a totally different architecture form SKADE produces random predictions when the N- and C- termini are removed and exactly the same AUC as the “standard” version when it predicts sequences to which the central region has been removed. We added this second experiment to the main text, highlighting it in red.

As you suggested, we ran some experiments with a non-redundant training set. The original training set contains sequences with less than 90% of sequence identity. In our test, we ensured a much stricter threshold, specifically on the N- and C- termini: first, we removed the central region from the sequences in the training set (20-80 percentile of length) and than we clustered them with BLASTCLUST, at 20% of sequence identity and 90% of coverage, obtaining 47528 clusters. We selected only one (full) sequence per cluster and we trained our model on this non redundant dataset, testing it on the Test set in the usual way. With this procedure we obtained an an AUC of 81, very similar to the AUC presented in the paper.

The train and test sets have been engineered to share less than 30% sequence identity (Rawi et al., 2018, Khurana et al., 2018) using CD-HIT, but we found that this definition of redundancy is less stringent than finding homology between the two sets with BLASTP, which is a local alignment tool. By applying this more stringent settings with BLASTP, we obtained a training set containing 38243 sequences with less than 30% sequence identity with respect to the Test set. Training on this smaller dataset and predicting the Test set, we obtained an AUC of 81, which is almost identical to the one obtained with “standard” train-test settings used in (Rawi et al., 2018, Khurana et al., 2018).
The corresponding attention plot for this new settings with more stringent redundancy threshold is shown in the following figure, which is analogous to Fig. 2 in the manuscript. From this plot it appears that even when we apply more stringent settings for the detection of similarities between the training and the testing sets, the relevance of the N- and C- termini is conserved.

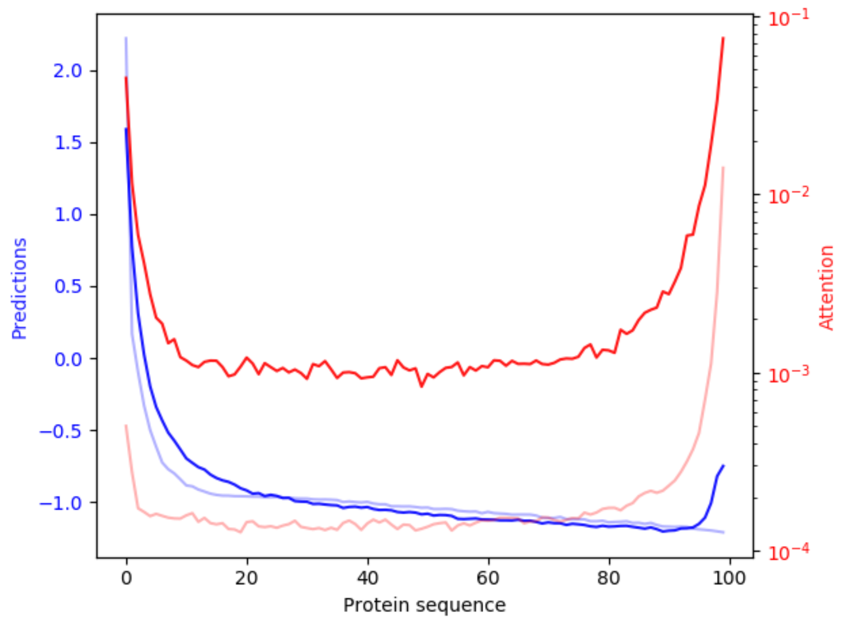

In the data reported for SKADE analysis of the CAMSOL mutation set, are all the mutations that affect solubility in the NT and CT regions - as might be expected given the results of Fig. 2?
Similarly, the synergistic mutations in later figures, are they also in NT and CT regions?

To address this point, we added a new figure (see Suppl. Fig. S7) showing how the mutations in CAMSOL are distributed over the protein sequences. These mutations are more or less uniformly distributed over the sequence, or at least do not have a “preference” for the N- or C- termini.
In Fig. 3 and 4 we investigate respectively **every** possible mutation and every possible pair of mutations, meaning that most of the variants will be mapped on the “central” region of the proteins.

We must also specify that although the results shown in Fig. 2 regarding the relevance of the N- and C-termini for the prediction show **the mean** attention values, meaning that single proteins may differ from this behavior (see for example Suppl. Fig. S3-5). Moreover, to predict the effect of mutations on the solubility of the protein, we compute the difference between the wildtype and the mutant protein. This means that, even if the change in the predicted solubility caused by a single mutation is small, the sign of the change in solubility (Delta(S)) will be predictive. In this way, even mutations causing a very small change, due to the fact that the model pays little attention to the central region of the protein, will be able to influence the final Delta(S).

Reviewer #2: The paper presents a novel computational approach to predicting protein and peptide solubility from amino acid sequence alone SKADE. The model behind the method is a Recurrent Neural Network with soft self-attention and dot-product score function, built using Gated Recurrent Units. It is implemented in Python using Pytorch framework and supplies a pre-trained network for immediate applications.
SKADE is a great example of a neural network design without explicit feature engineering. The resulting model is comparable in performance or outperforming (depending on metrics) state of the art methods such as PaRSnIP and DeepSol that rely on knowledge-based and engineered features. The distributions of weights and scores along protein sequences suggest that N- and C-termini are most important determinants of protein and peptide solubility.
In addition, SKADE is able to identify mutations that increase or decrease the overall solubility of the protein, making it a useful and fast method for scanning mutagenesis, not limited to single mutations. Testing the effects of protein truncation on solubility is also possible with SKADE.

Major:

1. Although the model provides per-residue attention values and scores, interpretability of the methods remains limited. The only conclusion is that N- and C- termini are more important, but that is about it. Besides, these are the output layers of the network, what is inside is still a “black box” in terms of interpretability, I suggest deemphasizing this point in abstract and conclusions. The lack of correlations with biophysical properties contradicts this conclusion.

We agree that the interpretability of our model relies mainly on determining “where” the model focuses its attention, and we clarified this in the manuscript, toning down our statements (changes highlighted in red).

Nevertheless, we believe that the conclusion that ML method(s) find the N- anc C-termini to be relevant for protein solubility is indeed a central aspect of this paper, indicating that even our (limited) attention-based notion of interpretability can lead to interesting conclusions. For example, we showed that also DeepSolS1 bases its predictions on the N- and C- termini, even though this behavior was not previously known, because the methods developed so far did not focus **at all** on the interpretability.

We do not agree with the observation that the lack of correlation with the propensity scales should be considered as a negative aspects of our model. On the contrary, we think that biophysical propensity scales are a quite simplistic way to quantify “average” properties of aminoacids, while in this study we are investigating complex protein-folding-related processes, which are sensitive to many factors including the *specific* local sequence environment to which each residue is exposed in the protein sequence *and* in the native structure.

In relation to this, we showed that the solubility profiles predicted by our model are to a certain extent predictive for experimentally determined beta-amyloidosis aggregation patches in the protein sequences, which is a quite complex task to be solved by bioinformatics approaches (see the generally low MCC values obtained by state of the art methods in Table 2). Without being trained to solve this task, our model performs similarly to methods specifically designed and trained for detecting aggregating regions, meaning that the model is able to grasp something that goes beyond the simple “average” values of the 20 residues encoded in the biophysical propensity scales. (Propensity scales alone are a very bad predictor for aggregating regions in proteins).

2. It is quite surprising to see that there is no correlation of attention and solubility profiles to any physico-chemical and/or structural properties of amino acids. Particularly, because these features work quite well in other solubility prediction methods. I think Table 1 in supporting information files does not comprehensively cover the biophysical features, for instance aggregation propensity, SASA. Besides, it is not clear how the correlations were calculated. Could the lack of correlation be related to scalability of attention/profile with sequence length? I.e. values of a short protein could be incomparable to values of a large protein.

We think that propensity scales just encode average characteristics of aminoacids. These values do not take into account the huge variety of **specific sequence environments** in which the residues are placed in the protein sequence and (spatially) during the protein folding process. Otherwise, these scales alone would be extremely good predictors for many structural bioinformatics tasks. Instead, since their performance were unsatisfactory, the community started developing ad-hoc ML models, sometimes using scales as inputs.
(Related to this common practice, recently we showed that even the use of propensity scales as inputs to ML method has likely no strong justification, since one-hot-encoding or randomly generated scales produce extremely similar performances on various archetypal tasks https://www.nature.com/articles/s41598-019-53324-w))

For what concerns the mathematical reasons for the lack of correlation, we do not think that it can be due to scaling problems, because we used the Pearson's correlation as correlation measure in Suppl. Table S1. (We have changed the text to explicitly mention the correlation.) Also other correlation measures (e.g. Spearman) give very similar results.

We think that the fact that the solubility profiles produced by our model do not clearly correlate with "trivial" properties of aminoacid is an indication that indeed our model is doing what would be expected from a non-linear model, meaning that it is learning complex patterns from the data. Such patterns cannot be easily summarized by a propensity scale *alone*. If our predicted solubility profiles had a strong correlation to, for example, the hydrophobicity of the aminoacids, it would mean that the optimal non-linear boundary learned by the model would just collapse to a basic properties of aminoacids such as their hydrophobicity.

For what concerns SASA, we show that SKADE's solubility profiles do not correlate with Relative Solvent Accessibility (RSA) predictions from an in-house tool we have. This is a NN-based supervised method that is unpublished because it gives performances similar to the state of the art RSA predictors, but without introducing particular novelties in its approach. We thus use it as part of in-house bioinformatics pipelines.

For what concerns the aggregation propensity, we show that notwithstanding the absence of correlation with propensity scales, the solubility profiles predicted by our model have an intriguing predictive power for **experimentally determined aggregation patches in proteins**, which is a quite complex prediction task. In our view this indicates that although the model does not correlate with single-aminoacid-based trivial biophysical aspects, it is able to detect biophysical patterns that relate to more complex emergent behaviors such as the beta-amyloidosis aggregation.

3. According to neural network architecture it seems that the Embedding for converting 20 amino acid categories into a 20-dimensional tensor acts simply as a One Hot encoder. If there is any actual embedding that is learnt during training could you visualize the resulting amino acid similarities? Otherwise I suggest renaming embedding to encoding.

We added a 2D visualization of the learned embeddings as Suppl. Fig. S5. This figure show the scatter plot of the 2 first principal components of a PCA computed over the 20-dimensional embedding space. During training, the embedding L2-regularized, so they are not sparse. We provide the exact values as separate supplemental .csv file.

4. Python 2.7 is not acceptable by any means. This Python branch reached end of life on January 1 2020, is no longer supported. Moreover, is currently being removed from many computer systems for security concerns because it is unsupported. See https://devguide.python.org/#status-of-python-branches . It looks like there should not be any reasons not to update the code to Python 3.

We have now rewritten the predictor in python 3.6 . (Pytorch does not work on python 3.8)

5. Please format code according to PEP8 https://www.python.org/dev/peps/pep-0008/ Automatic conversion with autopep8 --aggressive could be used, for instance. Note that there is a lot of imported but unused modules, variables, and other issues that a lint program could fix, please also run pylint to

get rid of the software issues.

We have now ensured the PEP8 compliance of our code with autopep8 and we removed the unused modules.

Minor:

1. There is an imbalance between soluble and insoluble classes, since you have not used weighted sampling. To what extent do you think this imbalance affected the results? I suspect this could be the reason behind performance gap with respect to positive/negative class prediction separating SKADE and PaRSnIP.

The training set contains ~30k positive and ~40k negative samples, and the imbalancement is thus not striking. The test is perfectly balanced (1001 positives and 1001 negatives).
In this settings, we do not think that imbalancement is an issue. The gap in performance with PaRSnIP is more likely explained by the fact that, while SKADE uses only the protein sequence as input, PaRSnIP uses different kind of information, including predictions from third-party tools (Secondary Structure, Relative Solvent accessibility, etc.). We chose not to use such features because we are interested into end-to-end differentiable pipelines, that can be more useful in the protein optimization context.

2. The details of model convergence should be provided in supporting information. I could see that tensorboard was used in the program, so it should be possible to include those plots.

Tensorboard plots have a resolution which is below the publication standards. We provide the plot showing the model convergence here nevertheless.

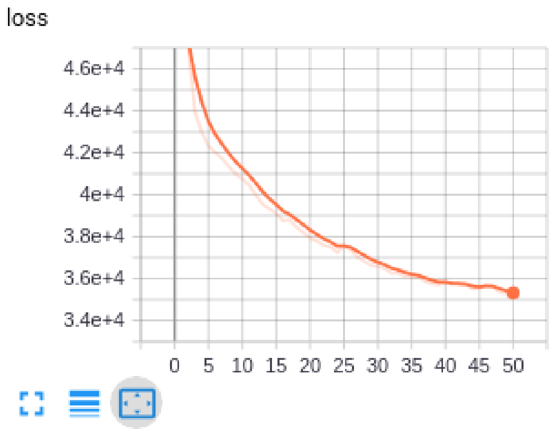

3. Line 98, why i<0 ?
Thanks for noticing this error, we changed it with i = 0.

4. Line 298: According to uniprot the protein should be 104, not 103 residues long https://www.uniprot.org/uniprot/O26734

We are using the sequence provided as training and test sets in PaRSnIP (https://academic.oup.com/bioinformatics/article/34/7/1092/4562500). These sequences may have small differences with respect to the current Uniprot sequence, due to 1) the continuous update of Uniprot and 2) the fact that they were originally taken from a different source.
Moreover, the ID used to identify the proteins in these training and test sets are not directly mapped on Uniprot Ids, and we thus **manually searched the corresponding gene** using homology search methods (BLASTP). Similar differences occur when comparing the sequence of the same protein obtained from NCBI or Uniprot databases.

For what concerns O26734, we determined the correspondence between this Uniprot entry and the entry number 18 of the test set with BLASTP. From the BLAST results, the O26734 (Y637_METTH) Uniprot sequence has 99.04 sequence identity with the entry 18 of the test set, with an E-value of 3e-71. Even though there are small differences, we can cleary state that O26734 (Y637_METTH) is the corresponding protein (and gene) for this sequence in the dataset.

5. The equation in line 300 is not clear, were the pairs of mutations generated including two mutations on the same position? Shouldn't it be something like 104x19x19x103/2?

Thanks for noticing this issue: for a sequence of L residues we evaluate all possible pair positions excluded itself, which are L(L-1)/2 (the upper triangle of a square matrix). Given the fact that for each position we have 20 possibile instances (including the wild type), when L=103, as in our case, the correct number is (103x102x20x20)/2.

6. Current Pytorch v 1.3.1, version was 1.0.1 used in the paper. Model needs to be recalculated

The current stable version of pytorch is the 1.4. The point is that pytorch versions are continuously updated, and it is not possible to provide a model that is always at the latest version. Versions of pytorch above 1 do not have breaking changes, and the stored models work interchangeably. (For example the final model in the repository has been actually trained and serialized on the 1.3 version, while the model development was originally made on the 1.0.1 version.)

To solve this issue once for all, we have now **serialized the actual weights** of the NN instead of the python object containing the NN. This is as version-independent as it gets, since they are just weight matrices that can be loaded in any future version of pytorch.

7. Please submit the model to kipoi.org repository, that would make it much easier to run it

We are surely thinking about making our model more accessible in the near future, either through a webserver or uploading it to a repository such as kipoi.

8. Peptide N- and C-termini are effectively charged residues in terms of their physico-chemical characteristics affecting solubility, i.e. Ala is hydrophobic, but will have a charge on the N-termini. However, it is not treated/encoded in any way different than any other amino acid. Was the reasoning behind it to an unbiased representation in the model or something else?

The unbiased representation of the sequence within our model was certain one of our greatest concerns in the development of SKADE: the aim of this work was to provide a simple and straightforward model able to predict the protein solubility. To do so we chose to use just the protein sequence as input, avoiding the computation of any other possibly relevant feature (e.g. Secondary Structure, Relative Solvent Accessibility, etc.), including local charge. This indeed is also a way to minimize the possible biases that could be introduced by more complex representations, problem that we have explored in one of our previous papers (https://www.nature.com/articles/srep36679).

9. What is BAC in table 2?

BAC is a acronym for Balanced Accuracy, namely the mean of Sensitivity and Specificity. We have now update the “Validation procedure and performance evaluation” section including this term.

10. Line 65, correlation is reported as AUC, which is not convenient

We have rephrased the entire sentence.

11. Line 319: “my” > “by”
We fixed that, thanks for noticing.

12. In my opinion it is better to show dependence of synergetic effect on spatial distance directly as a scatter plot to emphasize on the correlation, particularly in proximity

The correlation is between spatial distance, sequence separation and synergistic effects. For this reason, we think that the plot in Fig. 5 of the paper summarizes well all the 3 aspects. We nevertheless added two more plots as Suppl. Fig. S8, showing the plot in Fig. 5 as scatter plot (as you suggested).

13. In Figure 3 according to heatmap the first WT residue in O26734 should be V (the only white shaded residue), however this is not the case. What gives?

As mentioned before, the sequences in the training and test set used in PaRSnIP (and previous methods) do not derive directly from Uniprot or they derive from previous versions of it. This causes small differences between the current Uniprot sequence and the one present in the dataset. To retrieve the correspondence between the sequences in the test set and Uniprot ids, we used BLAST. From the BLAST results, the O26734 (Y637_METTH) Uniprot sequence has 99.04 sequence identity with the entry 18 of the test set, with an E-value of 3e-71. Even though there are small differences, we can cleary state that O26734 (Y637_METTH) is the corresponding protein (and gene) for this sequence in the dataset.
